# Genetic Approaches to Study Plant Responses to Environmental Stresses: An Overview

**DOI:** 10.3390/biology5020020

**Published:** 2016-05-17

**Authors:** Khaled Moustafa, Joanna M. Cross

**Affiliations:** 1 Conservatoire National des Arts et Métiers, Paris 75003, France; 2 Faculty of Agriculture, Inonu University, Malatya 44000, Turkey; joanna.cross@inonu.edu.tr

**Keywords:** plant stress tolerance, environmental stress, plant adaptation, plant stress response, gene expression profiling, gene expression study, DNA arrays, microarrays, next generation sequencing, NGS

## Abstract

The assessment of gene expression levels is an important step toward elucidating gene functions temporally and spatially. Decades ago, typical studies were focusing on a few genes individually, whereas now researchers are able to examine whole genomes at once. The upgrade of throughput levels aided the introduction of systems biology approaches whereby cell functional networks can be scrutinized in their entireties to unravel potential functional interacting components. The birth of systems biology goes hand-in-hand with huge technological advancements and enables a fairly rapid detection of all transcripts in studied biological samples. Even so, earlier technologies that were restricted to probing single genes or a subset of genes still have their place in research laboratories. The objective here is to highlight key approaches used in gene expression analysis in plant responses to environmental stresses, or, more generally, any other condition of interest. Northern blots, RNase protection assays, and qPCR are described for their targeted detection of one or a few transcripts at a once. Differential display and serial analysis of gene expression represent non-targeted methods to evaluate expression changes of a significant number of gene transcripts. Finally, microarrays and RNA-seq (next-generation sequencing) contribute to the ultimate goal of identifying and quantifying all transcripts in a cell under conditions or stages of study. Recent examples of applications as well as principles, advantages, and drawbacks of each method are contrasted. We also suggest replacing the term “Next-Generation Sequencing (NGS)” with another less confusing synonym such as “RNA-seq”, “high throughput sequencing”, or “massively parallel sequencing” to avoid confusion with any future sequencing technologies.

## 1. Introduction

During the last few decades, high-throughput analyses of DNA and RNA have been developed as powerful approaches in plant, animal, and human genetics. Profiling gene expression in a given tissue or under an experimental condition, such as responses to environmental stresses, is a fundamental prerequisite to understanding how functional networks operate in living systems. Gene expression profiling methods can be divided into qualitative or quantitative, absolute or relative, and carried out *in vivo* or *in vitro*. To probe the transcript levels in any particular or comparative biological context, a number of molecular approaches have been developed with different scalability levels of input and output. The most common of these approaches are Northern blot, RNase protection assay (RPA), differential display of mRNA by PCR (DD-PCR), serial analysis of gene expression (SAGE), DNA arrays, real-time quantitative PCR (qPCR), and high-throughput sequencing (or next-generation sequencing (NGS)). Each of these approaches has inherent advantages and disadvantages, relating mainly to reliability, scalability, throughput, technical specificity, cost, and difficulty. All of them are based on molecular hybridization (between complementary DNA sequences) or DNA sequencing (synthesis of DNA complementary strands). Various technical modifications are frequently suggested to improve these methods and answer specific purposes and applications. Here, we concisely compare and highlight the principles, advantages, and disadvantages of the key genetic methods used in gene expression profiling in response to environmental stresses or any other specific developmental or conditional stage. Beyond the scope of this snapshot are the technical and procedural details, for which readers can refer to specialized lab manuals or references given in the bibliography section that report on each approach individually.

## 2. Northern Blot

### 2.1. Setup and Use

The Northern blot is a molecular biology approach used to look for specific populations of RNAs in a mixture of total RNAs. It was developed in 1977 by James Alwine [1] and is still considered the gold standard in molecular biology research for studying the expression level of a particular gene or confirming the output of another technique. Recent reports demonstrate this versatility, e.g., analyze the APX (ascorbate peroxidase) gene’s expression profiling in cotton under oxidative stress [2] and the cotton metallothionein GhMT3a gene under different abiotic stresses [3], the heat stress-responsive TaMBF1c in heat tolerance in wheat [4], the plastid ribosomal protein S5 in cold stress tolerance in Arabidopsis [5], and Fe-chelatase (FeCh) against oxidative stress [6].

### 2.2. Principle

The method is based on the ability of a nucleotide strand to bind to its complementary strand. The name Northern blot was given in reference to the Southern blot [7], which uses the same principle albeit with DNA. Experimentally, total RNAs are extracted and separated according to their sizes on an agarose gel. Next, RNAs are transferred and fixed onto a nylon or cellulose membrane. A probe composed of the reverse complement of the RNA sequence of interest (cDNA) is synthesized with detectable nucleotides based on radioactive or nonradioactive labeling [8]. The latter solution is then mixed with the membrane at a given temperature. The probe specifically binds to its target RNA during the hybridization process. Binding specificity depends on the temperature. The bound probe is then detected by radio- or fluorography after washing off all the unbound mixture. Probe-target binding rates are proportional to target RNA amounts up to a certain level. Hence, the method is used to detect changes in the expression levels of a target gene in a particular tissue, cell type, or biological samples under given environmental conditions (for example, stressed *vs.* non-stressed plants).

Since its inception, many modifications have been suggested to optimize Northern blot outcomes and reliability. Among these modifications are vacuum-blotting, RNA-transfer visualization, and ultraviolet fixation of the RNAs [9]. These procedures increase the sensitivity of the approach while reducing its experimental duration. Some controls also improve the accuracy of the method. First, two different amounts of RNAs can be loaded onto the gel to check the proportionality of the bound probe with nucleic acid concentration. Second, it is important to check that RNA is loaded evenly into each gel lane. One or more housekeeping genes with fairly constant expression levels are typically used as a second probe, so that similar detection levels should be seen in each lane for these controls that will be used for data normalization.

### 2.3. Advantages

The main advantages of the Northern blotting approach are its relative simplicity, cost-effectiveness, reduced artifacts [10], and the possibility of providing valuable information about RNA identity, size, and abundance. Moreover, the method allows the detection of all expression variants of a particular gene (alternative splicing forms) in an investigated sample/condition. It is also sufficiently sensitive to detect small changes in gene expression level that other techniques such as microarrays cannot detect [11]. Moreover, the dilution and housekeeping gene controls ensure that the results are reliable. For this reason, the method is still used to validate the findings of other techniques, such as transcriptomic analyses. Finally, spotted membranes can be stripped of the probes and reused for hybridization with another target cDNA. Spotted membranes can also be dried and stored to be reused a long time later [12]. Another advantage is that non-radioactive Northern blot (*i.e.*, biotin-labeled probes) can be used to detect small RNAs in plants [13].

### 2.4. Disadvantages

The foremost limitation of the Northern blotting method is that only one gene is analyzed at a time, with an important amount of RNA and reagents required, making the approach time-consuming and relatively expensive for a large-scale analysis. Other pitfalls include degradation risks of RNAs by RNases contamination in work environments. However, this hurdle can be reduced by using RNase inhibitors (such as diethylpyrocarbonate (DEPC)) and by working carefully and quickly in a clean lab environment with sterilized materials.

## 3. RNase Protection Assay (RPA)

### 3.1. Setup and Use

The RNase protection assay was developed in the 1990s [14,15,16] as a standard laboratory approach to quantify mRNA levels of a gene of interest in a particular tissue, developmental stage, or point in time [17]. In plants, RPA was used, for example, to analyze the expression of the isopropylmalate synthase (IPMS) gene family in *Arabidopsis thaliana* [18]. It is still used in animal science even if recent examples are lacking applications in plant research. Moreover, the method was improved to analyze transcription rates from individual plant promoters [19].

### 3.2. Principle

The RPA method relies on the specific annealing of complementary DNA or RNA sequences, as does the Northern blot. A probe labeled with phosphorous isotopes (P^33^ or P^32^) [20] or with biotin [21] is synthesized for RNAs of interest. Next, total RNAs are extracted and combined with the probe. Ribonucleases are added to digest all single strands while leaving the hybridized products intact. The non-digested double-stranded RNAs represent the sequences that are complementary to the antisense probes corresponding to the genes of interest. RNases are then removed from the reaction by treatment with proteinase K. The double-stranded RNA populations (cRNA, mRNA) can be extracted using the phenol/chloroform extraction method [22]. The extracted RNA double strands are then electrophoresed in gel and detected with an autoradiography film.

### 3.3. Advantages

RNase protection assay is a highly specific and sensitive method to detect and measure the abundance of specific mRNAs in a sample of RNA mixtures [23]. Its level of sensitivity is about 20–50-fold greater than Northern blots [24]. RNase assay can be used to quantify the mRNA levels of genes of a high degree of sequence similarity (*i.e.*, members of gene families) [24]. It also tolerates a higher degree of RNA degradation without compromising the quality of the obtained data. In addition, the method offers the possibility of carrying out multi-probe assays [25] and mapping the transcription start sites and intron/exon junctions, or analyzing alternative splicing variants [26].

### 3.4. Disadvantages

The major drawback of the RNase protection assay is the lack of information on the transcript sizes [27]. In fact, it is the size of the synthetized probe that will determine the length of the protected fragment of the hybrid (double-stranded molecules). Another pitfall is that the antisense probe should be completely homologous to the targeted RNA to be protected from cleavage by the nucleases. Also, the assay is rather laborious and time-consuming. Some modifications have been suggested to increase the efficiency of the RNase protection assay and to decrease its time and cost without compromising its reliability. For instance, a faster and single-step RNA extraction method can be substituted for the proteinase K and phenol chloroform extraction [28]. 

## 4. Differential Display of mRNA by PCR (DD-PCR)

### 4.1. Setup and Use

Differential display (DD-PCR) was described in 1992 by Liang and Pardee [29] to measure the level of eukaryotic mRNA population in human cancer cells. It then was widely used to identify genes differentially expressed in different conditions or tissues. For example, it was used in *Citrus flavedo* tissues to identify and analyze the expression profiling of 98 low-oxygen-regulated genes [30] and 92 mango genes whose expression were altered in response to environmental stresses [31]. 

### 4.2. Principle

Similarly to Northern blot and RPA, the DD-PCR allows researchers to compare the gene expression levels in two tissues or experimental conditions or in the same sample/tissue under different conditions or over a course of time. The main idea behind the DD-PCR is to extract total RNAs from samples in different experimental conditions [32] and convert them into cDNA populations using degenerated primers matching the polyadenylate RNA tail with two extra nucleotides. A subset of the population is then amplified at low stringency conditions with degenerate primers and one or several arbitrary decamers. Amplification of a reduced population of cDNAs results in data sufficiently simple to be viewed on a gel. Bands of different intensities between samples correspond to differentially expressed genes. These bands can then be excised, re-amplified, cloned, sequenced, and reused to screen a cDNA library or as a probe in Northern experiments.

### 4.3. Advantages

DD-PCR offers several advantages, including the rapidity and sensitivity of the assay. Only small quantities of RNA are needed to compare several conditions or variables simultaneously, and to identify differentially expressed genes in more than one population [33].

### 4.4. Disadvantages

Although the DD-PCR technique is relatively simple, there are some strong biases related particularly to a high copy number of mRNA [34]. In addition, irreproducibility and false positive readings may result under the low PCR stringency conditions used for nonspecific primers [33]. Several variations, however, have been suggested to increase the specificity of the PCR amplification step while reducing the number of primers being used [35]. In particular, the cDNA population can be cleaved by a restriction enzyme and ligated to a known adapter sequence. Moreover, the use of polyT primers with an adapter sequence to create cDNAs directly results in a population harboring a known sequence [31]. Adapter sequences and primers used for cDNA amplification depend on the method alteration. For instance, long or anchored primers can be used [33], as well as decamers targeting the start codon [31].

## 5. cDNA Amplified Fragment Length Polymorphism (cDNA AFLP)

### 5.1. Setup and Use

cDNA Amplified Fragment Length Polymorphism (cDNA AFLP) pinpoints genes differentially expressed in different conditions or tissues. It was developed in 1996 [36] based on the AFLP approach [37]. Since then several improvements and variations have been suggested [35]. The method is widely used and cited in biological research. A search inquiry in Google Scholar, for example, with the term “cDNA AFLP” uncovered 367 hits for 2015. The number is reduced to 231 when the terms “plant” and “stress” are added. The method was used, for example, to identify genes differentially expressed in a drought-tolerant almond variety [38], to probe expression differences in bean varieties resistant and sensitive to wilt [39], to examine transcriptional regulation during cold acclimation in wheat [40], and to identify potential factors involved in cryopreservation in *Arabidopsis* [41]. 

### 5.2. Principle

The method involves the conversion of mRNAs to cDNAs. A subset is then amplified by PCR to produce a population sufficiently simple to be viewed on an acrylamide gel. This is achieved by the digestion of cDNAs with a rare enzyme cutter and then another restriction enzyme with frequent recognition sites. The fragments produced are then ligated to adapter sequences. A PCR reaction is next run with primers composed of the adapter sequence and two randomly chosen nucleotides. There are four combinations for each nucleotide, hence 16 possible primers at each end and 256 primer combinations. As a result, one set can amplify 1/256 cDNAs available or around 100 for a genome with 26,000 genes. The product is viewed on an acrylamide gel, and the primer matching the adapter for the rare cutting enzyme is labeled for visualization by radioactivity (original procedure) or fluorescence (modified procedure) [42]. A protocol for silver staining has also been developed [43].

### 5.3. Advantages

The method is efficient, relatively cheap, and technically simple using standard equipment. Moreover, no prior knowledge of the genome is required. The high stringency of the PCR conditions reduces false positives and nonspecific binding. As a result, the method is reliable and capable of detecting rare mRNAs. It is easy to view several controls for a given condition or time courses on a single gel. In addition, the number of mRNAs screened can be increased by performing several PCR reactions. The method offers flexibility and good mRNA coverage. Finally, the cDNA fragments produced are typically larger than 100 bp and therefore relatively easy to assign to a given gene.

### 5.4. Disadvantages

Gene-to-gene comparisons are not possible. All fragments of interest need to be cut from the gel and cloned for sequencing. In some cases, the tag is insufficient for determining the corresponding gene assignment. As a result, the identification of genes of interest is rather time-consuming. In addition, the best rare cutting enzyme cleaves slightly fewer than half the cDNAs while the frequent cutter cleaves around 90% of the population. Some fragments may not be PCR-amplified correctly, so a full coverage cannot be obtained. Two variations, however, have been suggested to improve genome coverage and to simplify the data produced. First, biotin can be incorporated during the step of cDNA synthesis by the use of a biotinylated oligodT primer [44]. After digestion with the rare cutter, the 3′ fragment is captured with streptavidin and digested with the second enzyme. The ideally unique fragment released is then ligated to adapters and amplified. The modification combined with labeling of the rare cutter adapter reduces the number of visible fragments produced by each mRNA. Second, high coverage expression profiling (HiCEP) improves coverage while decreasing false positives [45]. Thus, the biotin method is used with normal restriction enzymes and first adapter ligation before streptavidin purification. This ensures that a single tag of less than 700 bp is produced for each mRNA. PCR conditions are improved to reduce false positives, to ensure amplifications of all fragments, and to include all primer combinations. Three different florescent dyes are used for each group of primers for selective visualization. Products are separated by capillary electrophoresis. Coverages of 80% are typically reached, with 100% being possible.

## 6. Serial Analysis of Gene Expression (SAGE)

### 6.1. Setup and Use

Serial analysis of gene expression (SAGE) is a powerful gene expression profiling method that can be used to characterize the transcription levels of thousands of genes. It was originally developed in cancer research to identify new pancreatic transcripts corresponding to short diagnostic sequence tags of 9–10 base pairs [46]. Afterwards, it was successfully applied to the analysis of gene expression profiles in a wide range of conditions and organisms [47]. In plant research, SAGE has been used in a number of studies to investigate plant responses to biotic diseases [48] such as the cassava mosaic [49] or to abiotic stresses such as cold [50] and many others. More recently, ragweed pollen transcripts were probed to examine the effects of drought and/or high carbon dioxide (CO_2_) on gene expression [51]. Since its description in 1995, SAGE has gained robustness and substantial improvements. SAGE is now combined with next-generation sequencing methods (SuperSAGE). It was recently used, for example, to profile the photorespiratory genes in the bundle sheath cells of the C4 grass *Sorghum bicolor* [52].

### 6.2. Principle

The principle of SAGE relies on the representation of messenger RNAs (mRNAs) by a short tag and concatenation of these fragments to gain information on several mRNAs with one sequencing reaction. Thus, mRNA is extracted to synthesize double-stranded cDNAs that will be cleaved with anchoring enzymes (AE). The cDNA fragments downstream of the cleavage site are then disregarded and those upstream are ligated to two adapters in two separate reactions. The two reactions are then combined and cleaved again with an enzyme called tagging enzyme (TE) to release short sequence tags (of about ~10 bp) that will be ligated to form longer tags called di-tags. The di-tags will be amplified by PCR and ligated to form longer concatemers in a serial manner. The obtained concatemers can be cloned, sequenced, and analyzed with computer programs to identity the amounts of the individual tags present in the sample (a quantitative measure). The amount of specific tags relative to all the tags present in the library indicates the abundance of the transcript in the starting sample.

### 6.3. Advantages

SAGE offers the possibility of performing = simultaneous, quantitative analysis of a large number of transcripts while measuring absolute mRNA levels [46]. The main advantage of SAGE over other gene expression methods is that it does not require prior information on the genes of interest.

### 6.4. Disadvantages

SAGE requires a relatively high amount of input RNA and, therefore, cannot be utilized when the size of biological samples is a limiting factor (for example, scarce materials or tissues). An additional limitation is that the shortness of tags may prevent deep analysis of SAGE library data, limiting the applications field of the approach. Due to the low complexity of short tags, SAGE is prone to identify more than one transcript [53]. The approach is rather labor-intensive and expensive.

Several modifications have been suggested to overcome these limitations. Modified versions producing longer tags of 21 bp for LongSAGE [54] or even 26 bp for SuperSAGE [55] have thus been introduced. LongSAGE has been successfully used, for example, to identify novel fungal and plant genes involved in host–pathogen interactions [56,57]. Even so, the extension of SAGE tags to the 3′ cDNA end is still often necessary to unambiguously identify the matching RNA [58]. A third variant called MicroSAGE has also been developed [59]. This method requires 500- to 5000-fold less starting material than the classical SAGE technique and is simplified in all its steps, from RNA isolation to tag release. The number of additional PCR cycles is also reduced.

## 7. DNA Arrays

### 7.1. Setup and Use

DNA arrays (other related terms include microarrays, DNA chips, biochips, and macroarrays) are solid supports (glass slides or microbeads, or plastic membranes) spotted with a large number of DNA sequences to measure the expression levels of a large number of genes or entire genomes (transcriptomes) or to compare multiple genomic regions simultaneously for genotyping purposes. The first microarray was developed in 1995 using complementary DNA (cDNA) probes printed on home-made glass slides and used to measure the gene expression levels of 45 genes in *Arabidopsis* [60]. Since then, considerable advancements have been made in DNA array technologies that have extensively accelerated the throughput of biological analyses and discoveries, and inaugurated the “-OME and OMICS” era (the goal to quantify and characterize a total or subtotal of biomolecules). Using DNA arrays, genome-wide analyses of RNAs and DNA have become a standard approach in molecular biology and medical research. For example, comparative genomic hybridization (CGH) microarrays allow the study of entire genomes to reveal DNA variations between genomes. Recently, transcriptome analyses to study plant responses to different environmental stresses have been reported in response to biotic and abiotic stresses [61,62], cold [63,64,65], drought [66,67,68], salt [69], and heat [70,71].

### 7.2. Principle

**Figure 1:**
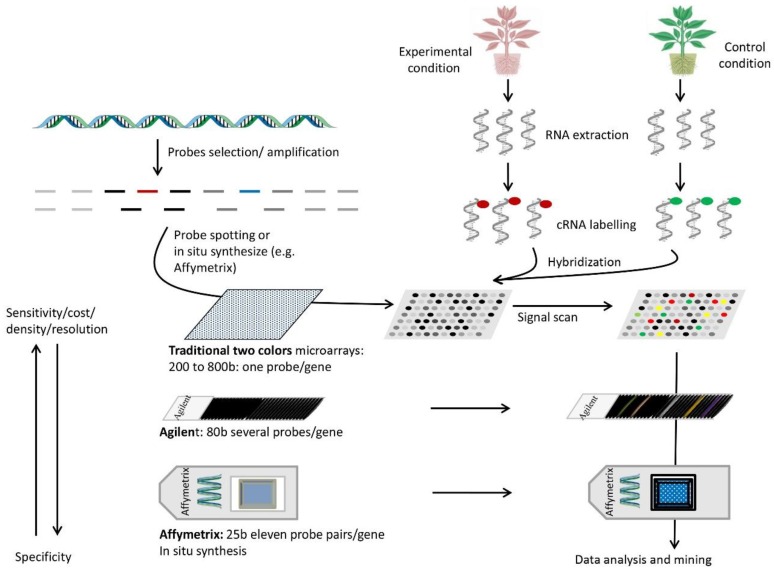
Steps and parameters of some types of microarrays used in gene expression profiling. Probes are selected for regular genome coverage or coding sequence studies (top left). They are synthesized directly on quartz slides or spotted on glass chips. Next, RNA is extracted from the samples of interest (top right), labeled with one or two dyes (depending on the microarrays type and labeling method), and hybridized to the probes (e.g., two colors for traditional microarrays or one color for Affymetrix or Illumina or other types). Samples are hybridized to separate chips if they are labeled with a single dye. The chip image produced by the label is scanned for each dye and the information combined to obtain expression differences between samples. Several parameters are important for microarray design. Longer probes are typically more sensitive but less specific. Affymetrix uses probe pairs, one perfect match and one a mismatch, to assess specific binding. Glass chips are cheaper but limited in probe density, while high density chips require a higher scanning resolution.

Figure 1 illustrates the general principle of key microarray methods. There are two main components in a DNA array approach: (1) the support (membrane or glass chip) on which probes are fixed, and (2) the preparation of RNA samples to be hybridized on the support. Several companies, such as Agilent and Affymetrix, provide custom-made supports spotted with a given number of probes. Supports can also be constructed in the laboratory for a defined purpose. For instance, a chip was constructed with sequences of grape genes potentially involved in dormancy [72]. Depending on the type of support being used (*i.e.*, beads and glass slides for high density, and plastic membranes for low-density analyses), hundreds to tens of thousands of probes can be printed on DNA arrays and performed in parallel or successively. The lengths of probes printed or synthesized *in situ* vary from short DNA sequences (for e.g., oligonucleotides ranging from 20 to 60 bases) to long DNA fragments (~200 to 500 bases).

The second component of DNA arrays consists of the choice of samples for RNA preparations. Indeed, gene expression profiling can be examined in comparative tissues or samples under different environmental conditions (for instance, control and stressed) or at variable growth stages. Total mRNAs are thus extracted from the tissues of interest and reverse-transcribed into cDNA populations in the presence of a labeling dye (for example, Cyanine 3 and Cyanine 5 for traditional two-color microarrays or an isotope P^32^ or P^33^ for membrane-based DNA arrays). In the case of two-color microarrays, the two labeled cDNA populations (experimental and control samples) are mixed for simultaneous hybridizations with their targets on the chip. In the case of membrane-based DNA arrays (macroarrays) or when one-color based microarrays are used (with biotin as a labeling dye), the hybridizations are processed separately with two separate sets of target supports, one for the control and the other for the experimental condition. The intensities of the hybridization probe target are then digitalized with specialized image processors to be normalized and compared. The hybridization values could be normalized using internal spots or global intensity on the array, and the data are compared to calculate fold changes between control and experimental conditions. The rates of fold change between samples indicate the sense of change and its magnitude.

### 7.3. Advantages

DNA arrays are powerful tools to unravel the molecular and genetic basis of biological processes on large scales that are unachievable using conventional approaches. Genome-wide approaches make it possible to analyze the transcription levels of hundreds to thousands of genes in parallel in an extremely short time compared to the past. The first and foremost advantage of DNA arrays is the rapidity, versatility, and high throughput capability of biological analysis (*i.e.*, thousands of genes or entire genomes are probed in one experiment within a day or so). Work that required months or years to be performed in classical approaches can be carried out within days, weeks, or months at most using DNA arrays, depending on the DNA array approach being chosen. DNA arrays are also reproducible and sensitive enough, though a confirmatory approach is often recommended to confirm the results obtained from DNA array methodology. Moreover, public array data websites are currently available, such as Genevestigator (https://genevestigator.com) and Bio-Array Resource (http://bar.utoronto.ca/), which provide array experiments data from many plant species in ready and easy to use formats.

### 7.4. Disadvantages

Despite their important advantages and versatility, DNA arrays present some limitations in terms of equipment, flexibility, and affordability, though important decreases in microarray costs are perceived due to the expansion of the microarrays market. However, most types of microarrays, particularly oligonucleotide-based microarrays (known as “GeneChips”), are still highly expensive and therefore not affordable for small to medium-sized labs. To carry out a microarray experiment, costly specialized reagents and equipment are required to print probes, label the target, perform the hybridization, quantify the hybridization signals, *etc*. In most cases, prior knowledge of genomes is also required to design specific probes or oligonucleotides, particularly in the case of Genechips. However, when such information is unavailable, alternative DNA methods can be used, such as cDNA microarrays. On the other hand, the use of commercial microarrays sometimes constrains researchers and limits their flexibility, since the manufactured microarrays are often centralized on manufacturers’ facilities and products. DNA arrays also suffer from a lack of standardization related mainly to normalization and statistical analysis toolkits, though substantial efforts have been made to set up a minimum of procedures and principles to standardize microarray experiments [73]. Other issues related to sensitivity, lack of reproducibility, and variability of the outcome of DNA arrays have been reported [74,75]. Additionally, the biological interpretation of DNA arrays’ data requires the combination of multiple genetic, physiological, biochemical, and bioinformatics backgrounds. However, despite these disadvantages, DNA arrays play major roles in modern genetic and bioanalysis systems. Substantial improvements and innovations are continually proposed to reduce DNA arrays’ caveats and increase their sensitivity, reliability, and affordability. However, with biotechnology progress, and as the cost of sequencing is continuously dropping, it is likely that DNA arrays will be replaced by sequencing methods or other new, more sensitive approaches in the future at least for some applications [76].

## 8. Real-Time PCR (or Quantitative PCR)

### 8.1. Setup and Use

Real-time PCR (RT-PCR), also known as quantitative PCR (qPCR or qRT-PCR), is an advanced variant technique of the traditional polymerase chain reaction (PCR) that has revolutionized the detection methods of RNA and DNA and substantially increased the applications and versatility of the classical PCR method. Real-time PCR is a robust method with a variety of applications such as gene expression quantitation, pathogen quantification (bacteria and viruses), molecular diagnosis, genotyping and gene variation detection assays, DNA damage measurement, and validation of data obtained by other techniques such as DNA arrays. The first documentation on real-time PCR appeared in the 1990s [77,78]. Since then, real-time PCR has been widely used in molecular biology experiments in humans, plants, and animals. Recently, it was used in the analysis of the ASR family in foxtail millet under drought/oxidative stress [79], the *Arabidopsis* pentatricopeptide repeat protein SOAR1 in response to salt, drought, and cold stresses [80] and the cotton GbSTK expressed in Arabidopsis in response to oxidative stress [81]. It was also reported that the heterogeneous expression of a new axanthin epoxidase gene (*MsZEP*) evaluated by qRT-PCR confers drought and salt tolerance in tobacco [82]. A novel highly selective qPCR was recently developed [83] for the quantification of human viruses, and could be used to investigate gene expression in plants too. 

### 8.2. Principle

Real-time PCR is based on exactly the same principle as the traditional PCR and comprises three steps: (1) denaturation of DNA template; (2) hybridization of specific probes to targeted sequences; and (3) primers’ extension to amplify or synthesize complementary DNA strands. The first step starts at about 94–95 °C for a few minutes to separate DNA double strands; the second at around 50–60 °C to allow hybridization between specific primers and their targeted DNA sequences; and the third step at around 68–72 °C to allow DNA polymerase to elongate the primers alongside their targeted template. The timing and temperatures for each step vary depending on different factors such as the template’s size, the percentage of cytosine-guanine (C-G), and the DNA polymerase being used [84]. These steps are generally repeated for 20 to 50 cycles.

A plot of DNA concentration over time shows a lag phase followed by an exponential phase then a saturation phase. During the exponential phase, the synthesized DNA molecules are proportional to the starting levels. A key feature of qPCR is that the amplified DNA is measured momently as the reaction progresses by the use of fluorescent markers that are incorporated into the PCR product. This is a new approach compared to the standard PCR, where the amplified product is detected only at the end. The result is that a special thermocycler coupled with fluorescence detection tools is necessary.

The real-time detection of the amplified amplicons can be visualized by two methods: (1) double-stranded DNA-intercalating dyes such as SYBR Green [85] that bind and fluoresce when bound to double-stranded DNA; or (2) fluorescent probes carrying a fluorescent reporter at one end and a quencher at the opposite end [77]. During the PCR cycles, the gradual increases in DNA concentration lead to increased fluorescence intensity, allowing the quantification of the DNA present in the sample.

### 8.3. Advantages

Over the past several years, the real-time PCR has become a primary tool for the quantification and detection of nucleic acids in biological samples, with great sensitivity, reproducibility, and specificity. Using qPCR, researchers can benefit from several important advantages including: the possibility of combining amplification and detection of target sequences in a single reaction, reducing post-PCR manipulations, displaying reaction progress in real time, precisely measuring the amount of amplicon at each cycle, and performing simultaneous throughput analyses in a short time. Although the lack of a systematic validation of reference genes is a serious pitfall for the reliability of results obtained by RT-PCR in plants [86], practical guidance encompassing key assay parameters was suggested to standardize qPCR studies and to help with the accurate design and optimization of qPCR experiments [87].

### 8.4. Disadvantages

Since the SYBR Green dye can bind to any double-stranded DNA, the major disadvantage of real-time PCR with this dye is that it might produce false positive amplifications (*i.e.*, binding to nonspecific double-stranded DNA sequences). However, to reduce this issue, a good primer design is required and melting curve analysis can also be monitored during the amplification cycles. Moreover, this inconvenience is less challenging when using fluorescent probes or dual hybridization probes; one probe carries a donor fluorophore at its 3′ end and the other an acceptor fluorophore at its 5′ end such as TaqMan, Molecular beacon, or Scorpions chemistry [84]. However, these alternatives are costly. Real-time PCR also requires special thermocyclers and commercial kits (reagents, dyes, and fluorescent primers) that are usually expensive for small to mid-range labs. It should also be emphasized that this method has strict requirements for careful assay design, optimization, and selection of stable reference genes. However, commercial and free *in silico* programs are available to help overcome these issues. For an accurate and optimized normalization of qRT-PCR data, multiple internal control genes [88] or the application of a model-based variance estimation approach for multiple genes [89] could be used.

## 9. Next-Generation Sequencing (NGS) 

### 9.1. Setup and Use

Next-generation sequencing (NGS) (also known as high throughput sequencing, deep sequencing, or massively parallel sequencing) is among the most recent and hottest technologies that have appeared on the market in the last decade. NGS technologies have revolutionized the biological and biomedical research for different purposes and applications in genomics, transcriptomics, and epigenomics, including whole genome genotyping, RNA sequencing (RNA-seq), mitochondrial genome sequencing, chromatin immunoprecipitation coupled to DNA microarray (ChIP-chip) or (ChIP-seq), detection of mutations and genetic disorders, clinical research, personal genome sequencing, and establishment of DNA libraries. The first description of NGS appeared in 1996 [90]. However, different NGS platforms and methodologies are currently developed and dominated by Illumina and Life Technologies. Recently NGS has been reported to identify differentially expressed genes, for example, in radishes under salt stress [91], in wheat under heat stress [92], in sugar beets under cold stress [93], in maize primary root tissues to investigate early transcriptome changes in response to moderate water deficit conditions [94], in barley to identify stress candidate genes in response to excessive boron [95], and in cotton to pinpoint genes responding to drought and salinity [96]. Interestingly, global transcriptome profiling analysis using RNA-seq reveals insight into cow saliva-responsive genes in alfalfa [97].

### 9.2. Principle

**Figure 2:**
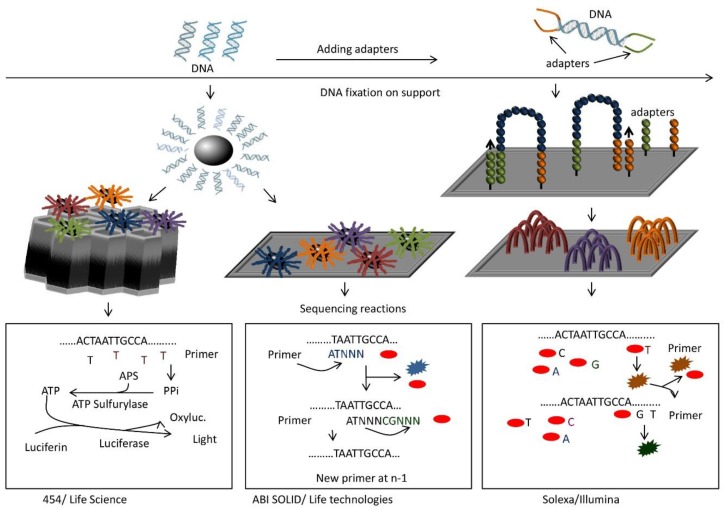
General principles of some types of “next generation sequencing” technologies. In all cases, cDNAs are sheared and ligated to adapter sequences (top). Then, each fragment is coated on a bead (left) and amplified. The bead with amplified fragments is inserted into a well (453/Life Science) or placed onto a slide (ABI SOLID/Life Technologies). Alternatively, fragments are hybridized to a flow cell carrying both adapter sequences (right), so that the fragments form bridges between two adapter sequences. After amplification and denaturation each strand forms a new bridge for amplification. This generates clusters of a fragment on the flow cell (right, Solexa/Illumina). Sequencing is done by synthesis for 454, synthesis with reversible terminators for Solexa, and ligation for SOLID. In the first case (bottom left), one type of dNTP is added at a time for primer extension. If the right dNTP is incorporated, pyrophosphate is produced and converted via two reactions into light and oxiluciferin. Light produced depends on the number of pyrophosphates generated and thus the number of a given dNTP incorporated. Solexa adds all four dNTPs at once for primer extension (bottom right). The dNTPs carry a reversible terminator sequence that prevents further extension, and a different fluorescent dye for each base. One base at a time is incorporated and determined by its fluorescence. Then the terminator and dye are cleaved and the cycle restarted. SOLID proceeds by ligation (bottom middle). Sixteen pentamers of all combinations of two bases and an additional three arbitrary nucleotides are added to the reaction. They are labeled with four different fluorescent dyes (one color for four combinations). The correct oligonucleotide anneals to the fragment next to the primer. A ligation reaction will connect the pentamer to the primer while all other fragments are eliminated. Incorporated group is determined by fluorescence. After cleavage of the terminator sequence and dye, the cycle restarts. After full synthesis of the complementary strand, all probes are stripped and the whole ligation cycle starts again with a primer at position n-1.

The principle of next-generation sequencing (Figure 2) is similar to the “previous” sequencing generation (Sanger and capillarity electrophoresis), based on the synthesis of DNA strands complementary to the strand to be sequenced. The main difference is that, in the first sequencing generations, only one DNA fragment is sequenced while in NGS the process is extended to millions of fragments in parallel. In Illumina sequencing systems, the DNA (or cDNA) to be sequenced is randomly fragmented and mixed with adapters that bind to 3′ and 5′ ends (library preparation). The adapted fragments are then captured on surface-bound oligos complementary to the adapters. The fragments are next amplified in the presence of fluorescently labeled deoxyribonuceotide triphosphates (dNTP) that will be incorporated into the DNA template by DNA polymerase during sequential cycles of DNA synthesis (complementary DNA strands). At the point of nucleotide incorporation, each nucleotide is excited and identified. The identified reads are then aligned to a reference genome and analyzed for mutations, single nucleotide polymorphism (SNP), RNA abundance, or phylogenetic analysis, *etc*.

### 9.3. Advantages

Enormous progress has been made in NGS technology in terms of throughput, read length, and speed. Using NGS, an entire genome can be sequenced within a single day or so. NGS allows comprehensive analyses of new genomes without prior knowledge of their sequences or their annotations while offering high sensitivity and accuracy to detect rare sequences and discriminate between closely related genomes. The NGS technology has also led to the development of a rapid genotyping-by-sequencing (GBS) approach that could have important impacts on genetic mapping strategies benefiting from a dense genome-wide distribution of markers [98].

### 9.4. Disadvantages

The main disadvantage of NGS technology is the relatively high cost, mainly for small and medium-sized budgets, though prices are becoming affordable due to competition between companies so that researchers can take advantage of some service providers at affordable costs. It also requires specific hard and soft infrastructure and special expertise to analyze and interpret the obtained data to extract the most meaningful information. Another limitation is that most currently available NGS platforms offer relatively short read lengths (~150–300 bases for Illumina and Life Technologies, and 450–700 bases for Roche454 sequencing), which is shorter than the conventional Sanger methods (~500–1000 bp) [99]. However, read lengths are increasingly extended with each sequencing generation, and several third-generation technologies are currently being developed, although they are neither widely available nor mature yet [100]. A further disadvantage of NGS (and to some extent DNA arrays) is that it is relatively complicated to do the downstream data analysis, though continual improvements and refinement are introduced.

An additional “semantic” inconvenience in our view is related to the name “next-generation sequencing,” as the term “next” refers to upcoming events. However, as the technology is already in use, and undoubtedly will be in use in the future, the term “next-generation” would seem inappropriate or at least confusing. Otherwise, what would we call a new sequencing method coming after the current next-generation sequencing, “next-next generation sequencing”? To avoid such vagueness, we suggest using other synonyms for NGS such as “high throughput,” “massively parallel,” RNA-seq, or “second generation” instead of next-generation.

## 10. Conclusions

Each method mentioned above has its own advantages and disadvantages, briefly summarized and compared in Table 1. There is no “best” approach in all its features; some advantages of one approach would outweigh some advantages of another, and some disadvantages of one method would be more critical than another and so on. Sequencing-based approaches, however, are more accurate for transcript identification with higher throughput abilities. Nonetheless, they are technically and financially challenging for many labs. On the other hand, hybridization-based methods are generally cost-effective and easy to use, but they are less sensitive at monitoring low copy number transcripts or overcoming non-specific hybridization issues. They are also targeted to a chosen set of genes.

The emergence of a new method may inevitably lead to the recommendation of eliminating older techniques. For instance, some scientists may advise a systematic use of qPCR rather than of a Northern blot on the basis that the new method is more accurate. However, most of the time the biologist wants to investigate whether gene expression changes not at all, significantly, or massively. This type of information is reliably and visually provided by a Northern blot at a cheaper cost. Moreover, a more sensitive RNase protection assay can be selected if sensitivity is an issue. Likewise, should one switch from microarrays to next-generation sequencing? Currently, NGS requires extensive bioinformatics analysis to obtain and analyze the data. In addition, the method is fairly recent and therefore still in the improvement phase. On the other hand, microarrays benefit from about 20 years of experience and innovation from companies and laboratories. As a result, the technique will likely provide faster, reliable results when good-quality chips are available. This, of course, may change in the future. Finally, should one perform a full transcriptomic study and forego techniques such as differential display and SAGE? The answer depends on the research question being investigated. Indeed, transcriptomics is performed to gain a view of cellular responses to various stimuli and/or to identify a set of genes differentially expressed under a given condition. In the second case, a few genes that are highly expressed and thus easy to study are selected for further functional and biochemical characterization. Methods such as SAGE and differential display may thus provide sufficient data at a lower cost.

In short, the choice of the appropriate molecular approach depends on many factors, including budget, lab facilities, overall cost efficiency, availability of wet and hard lab equipment (hardware and software), ultimate study goals, number of genes of interest, and the worker’s expertise on a particular approach. The ultimate aim of a research should be to answer a scientific question convincingly and not to merely produce massive amounts of well-polished data.

## Figures and Tables

**Table 1:** Comparison of some parameters of key molecular approaches used in gene expression profiling studies.

	Northern Blot	RPA	DD-PCR	SAGE	DNA Arrays	qPCR	NGS
No. of genes	low	low	medium	high	high	medium	high
Specificity	high	high	high	medium	medium	high	high
Targeted	yes	yes	no	no	yes/no *	yes	no
Scalability	medium	medium	medium	medium	high	high	high
Difficulty	low	high	high	high	high	medium	high
Cost	low	low	low	medium	high	medium	high

Abbreviations: RPA: RNA protection Assay, DD-PCR: Differential Display PCR, SAGE: Serial Analysis of Gene Expression, qPCR: quantitative PCR (real time PCR), NGS: next-generation sequencing. * depending on the type of DNA array and the availability of information on genomes.
